# Workplace violence towards workers in the emergency departments of Palestinian hospitals: a cross-sectional study

**DOI:** 10.1186/s12960-015-0018-2

**Published:** 2015-05-07

**Authors:** Motasem Hamdan, Asma’a Abu Hamra

**Affiliations:** School of Public Health, Al-Quds University, Jerusalem, occupied Palestinian territory; School of Public Health, Al-Quds University, Gaza, Palestine

**Keywords:** Workplace violence, Emergency departments, Risk factors, Causes, Effects

## Abstract

**Background:**

Workplace violence (WPV) in hospital emergency departments (EDs) is a common problem. The objective of this study was to assess the characteristics (level and type), associated risk factors, causes, and consequences of WPV against workers in Palestinian EDs.

**Methods:**

A cross-sectional study was conducted in 14 out of the available 39 EDs in Palestine: 8 from the West Bank and 6 from the Gaza Strip. Data were collected using a self-administered questionnaire between July–September 2013. Multivariate logistic regression models were used to examine risk factors associated with exposure to WPV.

**Results:**

A total of 444 participants (response rate 74.5%): 161 (32.0%) nurses, 142 (32%) physicians, and 141 (31.7%) administrative personnel. The majority (76.1%) experienced a type of WPV in the past 12 months: 35.6% exposed to physical and 71.2% to non-physical assaults (69.8% verbal abuses, 48.4% threats, and 8.6% sexual harassments). Perpetrators of physical and non-physical violence were mainly patients’ families/visitors (85.4% and 79.5%, respectively). Waiting time, lack of prevention measures, and unmet expectations of patients and their families are the main reasons for WPV. The multivariate regression analysis showed that younger personnel (OR = 2.29 CI 95% 1.309–4.036), clinicians (nurses and physicians) (OR = 1.65 CI 95% 0.979–2.797) comparing with administrative, and less experienced ED personnel (OR = 2.39 CI 95% 1.141–5.006) are significantly at higher risk of exposure to WPV (*P* < 0.05). Low level (40%) of violence reporting is evident, largely attributed to not enough actions being taken and fear of consequences. Violence has been shown to have considerable consequences for workers’ well-being, patient care, and job retention.

**Conclusions:**

Violence against workers in Palestinian EDs is highly common. The effects of violence are considerable. Multiple factors cause violence; however, EDs’ internal-system-related factors are the most amenable to change. Attention should be given to strengthening violence prevention policy and measures and improving incident-reporting system.

## Background

Violence against workers in the hospital emergency departments (EDs) is a common concern worldwide. EDs have been recognized as an environment with high potential for workplace violence [1-3]. ED workers frequently have to deal with violent patients who are under the effect of illness, pain, or intoxicated and to encounter visitors who are usually highly worried about their patients [4]. Studies have shown that workplace violence (WPV) has considerable implications for workers, for patients, and for the cost of services [1-3,5].

Despite the significance of the problem, there has been very little systematic research focusing on the issue in hospital EDs in the Middle East region [6,7], and Palestine is no exception. EDs of Palestinian hospitals provide initial treatment for a broad spectrum of illnesses, injuries, and life-threatening incidents. Similar to experiences from different countries worldwide, EDs in Palestine are highly utilized by patients seeking emergency services but also frequently accessed for routine care especially in the afternoons and during nights [8]. The difference, however, is that the EDs play a crucial role in responding to emergency care needs during the frequent episodes of violent conflict with Israel. For instance, the EDs of Gaza hospitals had to cope with a huge number of casualties and wounded, estimated to be more than 11 000, during the war on Gaza in August 2014 [9].

Kitaneh and Hamdan [10] assessed the prevalence of WPV among nurses and physicians working in all departments of governmental hospitals in the West Bank (WB). The study brought to attention the high prevalence of violence in the EDs (71%) and critical care units and showed that WPV is poorly documented and managed. Therefore, there is a need for understanding better WPV in EDs and the underlying factors in order to formulate appropriate prevention policy and measures.

The aim of this study is to get an in-depth view of the WPV experienced by ED workers in Palestinian hospitals. The assessment includes the level and types and the perceived causes and consequences of the WPV in EDs. Also, it reports on issues related to the source of violence, incident reporting, and hospital violence prevention measures and examines the demographic and occupational risk factors associated with exposure to WPV in EDs.

### Design

This is a cross-sectional study that investigated all types of violence against ED workers including physicians, nurses, and admission/registration personnel during the year before the survey.

### Setting

There are 39 hospitals providing emergency care in Palestine. Of these, the main 14 EDs were selected for study: these were 8 EDs from the West Bank (WB) and 6 from the Gaza Strip (GS). The selection took into consideration the representation of all regions (north, middle, and south) of the WB and Gaza Strip and equal mix of governmental and non-governmental sectors in each region, except that four EDs from the north of the WB were included due to the higher number of hospitals in that region. It is worth noting that governmental hospitals (18 EDs) are the main provider of emergency care services in Palestine [8], and the EDs in GS have a larger capacity and number of workers and patient visits than in the WB due to the violent conflict with Israel and frequent crises.

The study population consisted of the 596 personnel of the selected 14 EDs: 216 nurses, 201 physicians, and 179 administrative personnel, i.e. registration and receptionists. Due to the small size of the study population, all the workers in the studied 14 EDs who were available at the time of the study were targeted.

### Data collection instrument

Data was collected using a self-administered questionnaire. The instrument was adapted from our earlier study [10] that was conducted to assess WPV in Palestinian governmental hospitals and slightly modified to fit the objectives and context of this study in EDs. It included questions related to the characteristics of participants, the nature and frequency of exposure to violence in the past year, perpetrators, perceived causes, and impact on workers, as well as questions on hospital violence prevention measures. The survey items were prepared in the form of multiple choices and open-ended questions and were in Arabic. The questionnaire was reviewed by an expert panel that consisted of researchers and clinicians in order to ensure the content validity. The survey was then pilot tested in another hospital than those studied. Modifications on the wording were done to improve understanding of some questions by participants.

Definitions for all types of violence were given in the survey, mainly adapted from the ILO/ICN/WHO/PSI Joint Programme on WPV in the Health Sector [11]. Physical assault (PA) is the exposure to the deliberate use of force (such as hitting, kicking, slapping, choking, biting, or pushing) by any person and that may lead to physical, sexual, or psychological harm. Non-physical assault (NPA) includes threat, sexual harassment, and verbal abuse. Whereas, verbal abuse is any oral communication that negatively affects the dignity of somebody such as yelling, directing insult, nudging, or humiliating based on age, sex, race, colour, disability, language, religion, and economic or social status. Threat is the intent through use of words, gestures, signs, or behaviours to intimidate or harm the employee (physically or otherwise). And, sexual harassment is any unwelcome and non-reciprocal verbal or physical conduct of sexual nature such as insulting gestures, jokes, gifts, or offensive contacts.

### Data collection

Ethical approval was obtained from the institutional review board at Al-Quds University; also, permissions to conduct the study were granted by hospital administrations. Anonymous surveys were distributed together with a cover letter that included the aim of the study, survey instructions, and informed consent insuring participants confidentiality and voluntary participation. Data were collected in the period between July to September 2013.

### Data analysis

Data were entered and analysed using SPSS version 19. Descriptive statistics were performed to describe the characteristics of the participants and exposure to all types of violence. In the bivariate analysis, chi-square tests (*χ*^2^) were used to assess the associations between exposure to each of the physical and the non-physical violence and participant characteristics. Multivariate logistic regression models were used to examine risk factors associated with exposure (Yes/No) to any type of the WPV. The covariate gender, age, job category, education, hospital ownership, monthly visits to ED, experience in ED, and region were included in the model. *P* < 0.05 was accepted as statistically significant in all the analysis.

## Results

### Characteristics of the participants

Table 1 presents the characteristics of respondents. A total of 444 persons responded to the survey. The overall response rate was 74.5%: 80.1% for nurses, 65.8% for physicians, and 78.8% for the administrative personnel. Response rate in GS was 75.4% and 71.4% in the WB. Participants were mainly (70.5%) from GS and mostly work in governmental hospitals (72.2%). The majority were males (76.8%), half of them (52.2%) age above 30 years, and (67.6%) had a bachelor’s degree or higher. Clinicians formed 68.3% (nurses 36.3% and physicians 32%) of the participants, 57.7% worked in EDs with more than 9 000 monthly visits, and 56.2% had experience less than 5 years in EDs.

**Table 1:** **Participants demographic and professional characteristics (** ***n*** **= 444)**

**Characteristic**	***F***	**%**
*Region*		
West Bank	131	29.5
Gaza Strip	313	70.5
*Ownership*		
Governmental	323	72.7
Non-governmental	121	27.3
*Monthly ED visits* ^a^		
<3 000 visits	92	20.7
3 000–9 000 visits	96	21.6
>9 000 visits	256	57.7
*Gender*		
Male	341	76.8
Female	103	23.2
*Age*		
≤30 years	211	47.5
>30 years	233	52.5
*Job category*		
Physician	142	32.0
Nurse	161	36.3
Administrative	141	31.7
*Experience in EDs*		
<5 years	250	56.3
5–9 years	111	25.0
≥10 years	83	18.7
*Level of education*		
<Bachelor’s	144	32.4
≥Bachelor’s	300	67.6

^a^Source of data Ministry of Health [8] and direct contacts with hospital administrations.

### Prevalence of violence

A total of 338 participants (76.1%) reported exposure to at least one type of WPV in the past 12 months prior the study (Table 2). In specific, 35.6% reported physical assaults and 71.2% reported exposure to a type of non-physical assaults: 69.8% verbal abuses, 48.4% threats, and 8.6% sexual harassments.

**Table 2:** **Characteristics of violence**

	**Physical**		**Non-physical** ^**a**^	
	***F***	**%**	***F***	**%**
*Exposure to violence in the past 12 months*				
Yes	158	35.6	316	71.2
No	286	64.4	128	28.8
*Perpetrators of violence*				
Families/companions/visitors	134	85.4	233	79.5
Patients	16	10.2	42	14.3
Colleagues	7	4.5	18	6.1
*Need for treatment/care following violent incident*				
Did not need treatment	236	83.4	118	76.1
Treated myself	31	11.0	23	14.8
Needed treatment but did not receive	9	3.2	11	7.1
Treated by health professional (physician/nurse)	7	2.5	3	1.9
*Causes of violence*				
Waiting time	75	47.5	137	43.4
Lack of violence prevention methods	57	36.1	118	37.3
Unmet expectation of patients/families	56	35.4	100	31.6
Lack of medicines or needed services	28	17.7	59	18.7
Anxiety/fear/stress	26	16.5	73	23.1
Mental illness	23	14.6	47	14.9
Staff attitudes	16	10.1	33	10.4
Influence of illness/pain	12	7.6	42	13.3
Lack of people awareness	11	7.0	17	5.4
Influence of substance (drug/alcohol)	8	5.1	19	6.0
Do not know/others	6	3.8	12	3.8

^a^Exposure at least one time to either verbal abuses, threats, or sexual harassments; the prevalence of these were, respectively, 69.8%, 48.4%, and 8.6%.

With regard to physical assaults, 55.5% were pushing or pulling, 25.8% throwing furniture or instruments, 9% kicking or hitting, 3.2% using weapons, and 6.5% were other types. Of these assaults, 25% occurred in the morning, 38.5% in the afternoon, and 30.1% during the night shifts. Moreover, 61% took place in the waiting area, 22.7% in the examination/treatment rooms, and 16% in the EDs corridors.

Generally, violence whether it is physical or non-physical was mainly perpetrated by patients’ families/visitors, then by patients, and less frequently by work colleagues (Table 2). Sexual harassments largely (70%) came from the patient relatives/visitors, 22% colleagues, and 8% from the patients. While 76.1% of those exposed to violence reported that they did not need any kind of care, some of those exposed to PA and NPA (3.2% and 7.1%, respectively) needed care but did not receive it.

### Reason for exposure to violent action

Table 2 also shows the perceived factors that caused PA and NPA towards workers. These can be categorized into two groups. First, factors related to the EDs’ system, including waiting time as the key factor for violence (47.5% and 43.4%, respectively, for PA and NPA), lack of violence prevention measures (36.1% and 37.3%, respectively), lack of medicines or needed services (17.7% and 18.7%, respectively), and staff attitudes (10.1% and 10.4%, respectively). Second, patient- and their family-related factors including unmet expectations of patients/families (35.4% and 31.6%, respectively, PA and NPA), anxiety/fear (16.5% and 23.1%, respectively), the influence of mental illness (14.6% and 14.9%, respectively), illness or pain (7.6% and 13.3%, respectively), lack of people awareness (7.0% and 5.4%, respectively), and the influence of substance (drugs or alcohol) (5.1% and 6.0%, respectively).

### Violence prevention measures

The findings clearly showed a lack of violence prevention and management measures in the studied EDs: 67.3% indicated lack of violence deterrents (e.g. security personnel, camera, alarm, or communication system), 76.3% lack of violence prevention policy and procedures, and 82.6% lack of training on violence prevention and management.

### Violent incident reporting

Violence reporting was another relevant concern. While 40% of the victims reported incidents to their supervisors or to hospital managements, 39% indicated that the incident was not worth reporting. Workers do not believe that there is benefit in reporting because no actions are being taken (59%), there is fear of consequences such as revenge of assailant (17%), they feel shame over the incident (4.5%), there is a lack of proper reporting system or knowledge to whom to report (4.5%), and 1.5% for other reasons.

### Factors associated with violence

The results show (Table 3) that PA violence is significantly (*P* < 0.05) common among those working in EDs with the largest number of patient visits (>9 000 monthly visits) (40.2%) and among the younger workers (≤30 years) (41.2%). In comparison, NPA was significantly (*P* < 0.05) more prevalent among governmental hospital workers (75.9%), in EDs with the largest number of patient visit (78.9%), and among clinicians (78.9%), as well as among the younger age (76.8%) and less experienced staff members (70.8%).

**Table 3:** **Exposure to physical and non-physical violence by participant characteristics**

	**Physical**				**Non-physical** ^**a**^			
	***F***	**%**	***χ*** ^**2**^	***p*** **value**	***F***	**%**	***χ*** ^**2**^	***p*** **value**
*Region*								
West Bank	42	32.1	1.007	0.316	92	70.2	0.08	0.77
Gaza	116	37.1			224	71.6		
*Ownership*								
Governmental	122	37.8	2.469	0.116	245	75.9	12.65	<0.001
Non-governmental	36	29.8			71	58.7		
*Monthly ER visits*								
<3 000 visits	92	25.0	7.123	0.028	92	58.7	17.962	<0.001
3 000–9 000 visits	96	33.3			96	62.5		
>9 000 visits	256	40.2			256	78.9		
*Gender*								
Male	128	37.5	2.44	0.18	241	70.7	0.177	0.674
Female	30	29.1			75	72.8		
*Age*								
≤30 years	87	41.2	5.59	0.018	162	76.8	6.16	0.013
>30 years	71	30.5			154	66.1		
*Job category*								
Physicians	41	28.9	4.5	0.105	112	87.9	9.45	0.009
Nurses	65	40.4			116	72.0		
Administrative	52	36.9			88	62.4		
*Education*								
<Bachelor’s	58	40.3	2.047	0.153	99	68.8	0.609	0.435
≥Bachelor’s	100	33.3			217	72.3		
*Experience in ER*								
<5 years	90	36.0	4.66	0.097	177	70.8	7.34	0.025
5–9 years	46	41.4			88	79.3		
≥10 years	22	26.5			51	61.4		

*χ* ^2^: Pearson chi-square.

^a^Nonphysical violence includes threats, verbal abuse and sexual harassment.

Table 4 shows the risk factors associated with exposure to WPV in general. We can see that WPV in general is significantly more prevalent among ED workers in governmental hospitals (80.2%) (*P =* 0.001); those working in EDs with a higher patient visit (*P =* 0.001); younger personnel (81.5%) (*P =* 0.007); clinicians, e.g. nurses and physicians (78.9%) (*P =* 0.032); and the less experienced (less than 9 years) (*P =* 0.042) (Table 4). After adjusting for significant (*P* < 0.05) independent predictors of WPV (ownership, monthly ED visits, age, job category, and experience) using a multivariate logistic regression model, the results showed that age, job category, and experience remained significant risk factors. In specific, the risk of exposure to WPV was 2.3 times higher for younger workers (≤30 years) (OR = 2.29 CI 95% 1.309–4.036), 1.7 times higher for clinicians (nurses and physicians) (OR = 1.65 CI 95% 0.979–2.797), and 2.4 times higher for the less experienced than for other workers (OR = 2.39 CI 95% 1.141–5.006) (*P* < 0.05).

**Table 4:** **Risk factors associated with exposure to workplace violence**

	**Bivariate analysis**				**Multivariate analysis**		
	***F***	**%**	***χ*** ^**2**^	***P*** **value**	**Adjusted OR** ^**a**^	**95% CI**	***P*** **value**
*Ownership*							
Governmental	259	80.2	10.748	0.001	1.14	0.392–3.319	0.809
Non-governmental	79	65.3			1.0	Reference	
*Monthly ED visits*							
<3 000 visits	58	63.0	14.120	0.001	1.0	Reference	0.086
3 000–9 000 visits	70	72.9			1.9	0.652–5.541	
>9 000 visits	210	82.0			3.476	1.043–11.585	
*Age*							
≤30 years	172	81.5	6.428	0.007	2.299	1.309–4.036	0.004
>30 years	166	71.2			1.0	Reference	
*Job category*							
Clinicians (nurse/physician)	239	78.9	3.975	0.032	1.654	0.979–2.797	0.06
Administrative	99	70.2			1.0	Reference	
*Experience in ED*							
<5 years	108	73.5	6.330	0.042	0.984	0.504–1.922	0.017
5–9 years	117	83.6			2.39	1.141–5.006	
≥10 years	113	72.0			1.0	Reference	
*Gender*							
Male	257	75.4	0.467	0.294	1.0	Reference	
Female	81	78.6			1.371	0.77–2.443	0.284
*Region*							
West Bank	99	75.6	0.031	0.475	1.0	Reference	
Gaza	239	76.4			1.02	0.569–1.827	0.948
*Education*							
<Bachelor’s degree	107	74.3	0.389	0.305	1.0	Reference	
≥Bachelor’s degree	231	77.0			1.185	0.695–2.020	0.533

OR: Odds ratios, CI: Confidence interval, Reference: reference category in the logistic regression model, *χ* ^2^: Pearson chi-square.

^a^Adjusted for gender, age, job category, education, hospital ownership, monthly visits to ED, and experience in ED, and region covariates.

### Effects of violence

The reported most common effect of violence on exposures is provided in Table 5. About 74% of the victims of violence reported adverse consequences, mostly changes in the attitudes of workers especially among clinicians towards patients and their families (23.9%). For instance, 26.4% of physicians and 21.8% of the nurses indicated that they have minimized contacts with patients and their companions post-violence and 13.6% and 14.5%, respectively, minimized the time of patient care, as well as 11.8% and 8.2%, respectively, avoided taking decisions that might involve medical risks.

**Table 5:** **The most common effects of violence on different job categories**

**Type of effect**	**Physicians**	**Nurses**	**Administrative**	**Overall**
	***F*** **(%)**	***F*** **(%)**	***F*** **(%)**	***F*** **(%)**
Minimize communication, contact with patients/families	29 (26.4)	24 (21.8)	19 (23.5)	72 (23.9)
Hopelessness/disappointment	29 (26.4)	16 (14.5)	13 (16.0)	58 (19.3)
Minimize time of patient care	15 (13.6)	16 (14.5)	9 (11.1)	40 (13.3)
Fear and anxiety	4 (3.6)	16 (14.5)	10 (12.3)	30 (10.0)
Avoid taking decision that might involve medical risks	13 (11.8)	9 (8.2)	3 (3.7)	25 (8.3)
Feeling to take revenge	5 (4.5)	13 (11.8)	3 (3.7)	21 (7.0)
Feeling of guilt	0 (0.0)	2 (1.8)	2 (2.5)	4 (1.3)
No impact on me	15 (13.6)	14 (12.7)	22 (27.2)	51 (16.9)

*χ*2 *=* 31.574, *P* < 0.005.

Violence also has negatively affected the mental health and well-being of the workers in terms of expressed hopelessness/disappointment, fear, and anxiety (19.3%) as well as feelings of guilt (1.3%). Another important effect was the feeling to take revenge, reported by 7.0% of the victims. Physicians (26.4%) significantly felt hopelessness and disappointment more than other workers; in comparison, nurses (14.5%) reported a higher level of fear, anxiety, and feelings of guilt than other workers (*P* < 0.05). Overall, administrative personnel reported the least post-violence effects; 27.2% had no impact (*P* < 0.05).

We also used the intention to quit work in EDs as a proxy outcome measure to assess the impact of violence on workers. The analysis showed that those exposed to WPV in general were 3.5 times more likely to quit their jobs in EDs (OR = 3.48, CI 95 % 1.879–6.433, *P* < 0.001) than those who were not; this intention was 2.2 for PA (OR 2.18, CI 95% 1.21–3.90 *P* < 0.001) and 3.2 for NPA (OR 3.17, CI 95% 1.78–5.67 *P* < 0.001).

## Discussion

The study showed a high prevalence of WPV (76.1%) (35.5% physical and 71.2% non-physical) in the Palestinian hospital EDs. While PA was higher than reported in studies from the same region, however, the NPA was obviously less prevalent [6,12-15]. The prevalence of sexual harassments (8.6%) was also lower than reported from elsewhere [4,16-19]. Sexual harassment issue in health care institutions has not been adequately investigated in the Middle East region [18] because health workers, especially females, would feel reluctant to respond to studies due to the cultural sensitivity of the issue and the fear of being stigmatized.

Our findings, confirming previous evidence [2,6,12,15,20], showed that violence was mainly perpetrated by patient families or companions and patients themselves. The violence from colleagues (5.6% of PA, 6.1% of NPA) is worrisome. Co-worker violence has been attributed to job stress and low job satisfaction [10]. This would require interventions to promote a culture of respect between colleagues and adopting effective precautions to minimize the violent behaviour of staff members [16].

The bilateral analysis showed that physicians were highly exposed to non-physical (NPA 78.9%) compared with nurses or any other ED personnel (*P* < 0.05), but there was no significant difference in their exposure to physical violence. This agrees with a local study in all hospital departments [10] but disagrees with several studies [1,6,12,17] that showed nurses as mostly victimized. Kitaneh and Hamdan [10] have previously explained this, by linking it to the dominant cultural values towards the medical profession, considering physicians ultimately accountable for patient care, that frequently exposes them to violence from dissatisfied patients and visitors. Meanwhile, gender differences were not significant for all types of violence. This disagrees with several studies that reported more significant exposure of either the females [7,12,13,21] or the males [4,13]. It worth mentioning that violence against women in Palestine is not prevalent [22], and it is in fact a denounced behaviour.

In coherence with other studies [4,7,10,23,24], the results showed that younger and less experienced ED workers were more likely to experience violence. This is because senior personnel usually have more experience in recognizing and dealing with violent patients [4]. This demonstrates the need for educational programmes for junior ED personnel on preventing and dealing with violence [25]. Supported by evidence from regional studies [6,12], the results showed also that working in governmental hospitals is associated with higher exposure to violence than other hospitals. Perceptions of long waiting times due to the large number of Palestinian families benefiting from the public services through a governmental insurance scheme is a key factor. Another reason may be dissatisfaction of the patients and their families due to possible shortages of human resources and medical supplies in governmental hospitals.

In general, the two groups of factors causing violence against workers in Palestinian EDs (Table 2) are very similar to those reported in several studies [6,12,17,20,23]. To deal with the ED-related factors, hospital managements can work on decreasing waiting time through better utilization of resources and introducing a triage system to prioritize patients and to identify those who cannot wait long to be seen [26]. ED managements also need to ensure adequate medications and supplies and enhance deterring measures, e.g. security personnel, cameras, alarm, and communication systems, to protect workers from violence. In addition, there are several strategies that can be adopted to address the patient- and family/visitor-related factors. Experiences have showed that well-prepared ED education programmes can be an effective strategy to train workers on dealing with aggressive persons and protecting themselves against violence [17,25]. Hospitals can also ensure skilled personnel, e.g. social worker, to provide counselling to patients in EDs and help them to cope with their fear, anxiety, and stress [17].

Reporting of violent incidents is essential for ED managers to adequately understand the situation and consequently plan effective measures to reduce violence that occur. However, despite the high prevalence and serious consequences of violence on ED workers, underreporting of violent events is still widespread and common [2,6,12,16,23,27]. Our findings confirm the previous results that a common reason for underreporting is that workers perceive exposure to violence as “part of the job” or a minor event [10,16,23,28]. But, the most important barrier reported in this and other studies [15,28] is the lack of confidence that reporting will have any benefit for the reporters. It is crucial that hospital administrations follow up on reported incidents, take action against the perpetrators, and provide feedback to reporters. In addition to that, administrators and supervisors should provide support to the reporters and protect them against consequences that might arise, i.e. the perpetrators’ revenge.

Our findings are in line with available evidence regarding the negative consequences of exposure to violence. The most obvious implication was for patient care; a similar effect was reported by two earlier studies [6,21]. In addition, evidence showed that violence can have serious short- and long-term implications for the mental health and well-being of the exposed [16,20,29,30]. In fact, our findings showed that violence has led to psychological effects; among 30% of the exposed revealed fear, anxiety, hopelessness, and feelings of guilt. Violence was also found as a significant factor in personnel turnover in EDs. Majority of the physicians (78.3%) and nurses (78.7%) who had been exposed to violence indicated intention to quit work in EDs, which is much higher than reported elsewhere [4,6,16]. This possibly could complicate job retention and lead to shortages of qualified personnel in EDs [1,6,16]. Hospitals should provide care and social support to reduce the negative mental and physical effects and also to minimize the negative attitudes that might appear among ED staff towards work after workplace violence incidents [31,32].

### Strengths and limitations

The strength of the study is that it covered the main providers of emergency care in all the regions of the WB and GS. Moreover, the survey was anonymous and self-administered that probably made participants provide more valid responses especially to sensitive issues such as sexual harassment, besides eliminating the interviewer bias. It also had an adequate response rate for all types of ED works. However, we should acknowledge the possible reluctance of participants to report exposure to violence because of fear of stigmatization. Underreporting could also have happened due to the perception that violence is “part of my job”. Lastly, participants were requested to report about violence experiences back to 1 year prior to the study; this might have led to some recall biases.

## Conclusions

Violence against workers in Palestinian EDs is highly common. Health workers with direct contact, especially the young and least experienced, are the most exposed. Multiple factors cause violence; however, ED system-related factors are the most amenable to change. The immediate- and long-term psychiatric consequences of violence on ED workers are considerable. Moreover, the implications of violence for personnel retention is serious. All stakeholders, including the government, policy makers, services providers, and professional associations, need to work collaboratively to develop a national policy and violence prevention programme to tackle the roots of the problem in order to retain qualified human resources in EDs, maintain their well-being and productivity, and contribute to provision of adequate emergency health care services.

## Abbreviations

EDs: Emergency departments
GS: Gaza Strip
ICN: International Council for Nurses
ILO: International Labour Organization
MoH: Ministry of Health
NPA: Non-physical assaults
PA: Physical assaults
PSI: Public Services International
WB: West Bank
WHO: World Health Organization
WPV: Workplace violence
